# Editorial: Supramolecular Chemistry at the Interface of Environmental and Food Science

**DOI:** 10.3389/fchem.2021.680372

**Published:** 2021-04-30

**Authors:** Cheuk-Fai Chow, D. Amilan Jose, Pauraj Mosae Selvakumar

**Affiliations:** ^1^Department of Science and Environmental Studies, The Education University of Hong Kong, Tai Po, Hong Kong; ^2^National Institute of Technology Kurukshetra (NIT-Kurukshetra), Kurukshetra, India; ^3^Science and Math Program, Asian University for Women, Chittagong, Bangladesh

**Keywords:** supramolecuar chemistry, environmental, food science, chemical sensor and biosensor, catalysis & biocatalysis

Supramolecular chemistry has emerged as a valuable tool in the formulation of sensors to detect toxins in food (Gawley et al., 2002), sensors for environmental monitoring (Gale and Caltagirone, 2015; Ghosh et al., 2016), and catalysts to convert pollutants into reusable materials amongst other applications (Ishihara et al., 2016). The development and discussion of new sciences, techniques, and methodologies are essential if we want to bring these emerging technologies to fruition. This Research Topic mainly focused on the recent research at the interface of supramolecular chemistry and environmental science for applications in various fields such as degradation of environmental pollutants, detection of anions, detection of pesticides, and detection of food contaminants.

Five original research articles and two review articles by renowned academic researchers across the world have been collected. These articles will offer a comprehensive outlook for the application of Supramolecular Chemistry in the area of environmental and food science. We believe this Research Topic should attract chemical, biological, and analytical researchers and promote Supramolecular chemistry applications in various interdisciplinary research fields.

Dar and Sankar reported Ni(II) porphyrins-based receptor to detect environmentally essential anions and cations by supramolecular interactions. Toxic CN^−^ and F^−^ ions were detected via anion-induced deprotonation. On the other hand, metal ions such as Cu(II), Fe(III), and Hg(II) sensing were performed through chelation of CHO and NH. Manna et al. reported a sensitive colorimetric anion senor based on urea/thiourea receptor having a 4-nitrophenyl reporting unit. The nitro group was used as a chromophore and hence an effective probe for spectroscopic and visual discrimination of fluoride and dihydrogen phosphate in the testing solutions.

An exciting report to detect toxic organophosphorus pesticide, profenofos (PFF), was described by Chen et al. In this report, poly(styrene-co-methyl acrylic acid) based responsive molecularly imprinted polymer (PS-co-PMAA@PSMIPs) was prepared. PS-co-PMAA@PSMIP showed a good photo-responsive property and exhibited a linear sensing response to a wide range of PFF concentrations. Ultimately, the polymeric sensor was applied to determining PFF in spiked tomato and mangosteen fruits, and it resulted in a good recovery. Nao et al. reviewed different G4-based strategies to detect Ochratoxin A (OTA) which is the most common and toxic ochratoxins. It is important to note that ochratoxins are mycotoxins, and it causes serious illness through food contamination. In this systematic review, the authors firstly introduced the current status of OTA detection by chemical and biochemical methods and later discussed the advantages and disadvantages of G4-based methods for the detection of OTA.

Cucurbiturils (CBs) are versatile and robust supramolecular macrocyclic hosts to study the compound's catalytic activity. Ai et al. employed a recognition tunneling technique to investigate the conducting properties of melphalan@CB[7] and cucurbit[7]uril complex molecule junctions at molecular levels. The conductance of both Mel@CB[7] and CB[7] with different organic solvents and pH, found to be impacted by both the guest molecule and pH. It was confirmed that the conductance of molecular junctions would be affected by the solvents and pH, leading to investigating further with aqueous media. This report brings a new idea of electronic measurements of single molecular detection host-guest systems which are much more pH-responsive. Berta et al. investigated the supramolecular catalytic activity of CBs using MM/QM techniques and DFT calculations. Furthermore, the experiment was carried out to induce conformal changes, electrostatic changes of shielding effects by the highly polar aqueous environment. The MM/QM simulation techniques showed that the hydrogen bonding of the substrates has cooperativity in clear water. The results exhibit reasonable specificity and provide a broader opportunity to widen the application of supramolecular macrocycles as a catalytic host.

In a review article, Au elaborated the advancements of metal-organic frameworks (MOFs) to absorb environmentally unfriendly organic dyes which are extensively being used in the textiles, packing, paint, and color production-based industries. Since the excretion of these dyes directly to the environment has an adverse effect, purification of these from contaminated water is essential. The development of novel MOFs is one of the emerging ways to remove these dyes. In this review, the unique properties of MOFs, such as modifiable porous structures facilitating a high absorption rate, are discussed.

We believe that the articles and reviews published in this particular collection will help to develop Supramolecular Chemistry at the Interface of environmental and food Science. We appreciate all the authors for their significant contributions—also, our sincere thanks go to all reviewers for their constructive comments in evaluating the manuscripts.

## Author Contributions

All authors contributed equally to wrote, read, review the manuscript, and approved the submitted version.

## Conflict of Interest

The authors declare that the research was conducted in the absence of any commercial or financial relationships that could be construed as a potential conflict of interest.
